# Trimethylamine N-Oxide Response to a Mixed Macronutrient Tolerance Test in a Cohort of Healthy United States Adults

**DOI:** 10.3390/ijms24032074

**Published:** 2023-01-20

**Authors:** Kristen L. James, Erik R. Gertz, Catherine P. Kirschke, Hooman Allayee, Liping Huang, Mary E. Kable, John W. Newman, Charles B. Stephensen, Brian J. Bennett

**Affiliations:** 1 Department of Nutrition, University of California-Davis, One Shields Avenue, Davis, CA 95616, USA; 2 USDA-ARS Western Human Nutrition Research Center, 430 West Health Sciences Drive, Davis, CA 95616, USA; 3 Department of Population & Public Health Sciences, Keck School of Medicine, University of Southern California, Los Angeles, CA 90033, USA; 4 Department of Biochemistry & Molecular Medicine, Keck School of Medicine, University of Southern California, Los Angeles, CA 90033, USA

**Keywords:** trimethylamine-n oxide, meal response, mixed macronutrient tolerance test, cardiovascular disease, gut microbiota, precision nutrition, phosphatidylcholine, bile acids, metabolomics, metabolites

## Abstract

Plasma trimethylamine n-oxide (TMAO) concentration increases in responses to feeding TMAO, choline, phosphatidylcholine, L-carnitine, and betaine but it is unknown whether concentrations change following a mixed macronutrient tolerance test (MMTT) with limited amounts of TMAO precursors. In this proof-of-concept study, we provided healthy female and male adults (*n* = 97) ranging in age (18–65 years) and BMI (18–44 kg/m^2^) a MMTT (60% fat, 25% sucrose; 42% of a standard 2000 kilo calorie diet) and recorded their metabolic response at fasting and at 30 min, 3 h, and 6 h postprandially. We quantified total exposure to TMAO (AUC-TMAO) and classified individuals by the blood draw at which they experienced their maximal TMAO concentration (TMAO-response groups). We related AUC-TMAO to the 16S rRNA microbiome, to two SNPs in the exons of the *FMO3* gene (rs2266782, G>A, p.Glu158Lys; and rs2266780, A>G, p.Glu308Gly), and to a priori plasma metabolites. We observed varying TMAO responses (timing and magnitude) and identified a sex by age interaction such that AUC-TMAO increased with age in females but not in males (*p*-value = 0.0112). Few relationships between AUC-TMAO and the fecal microbiome and *FMO3* genotype were identified. We observed a strong correlation between AUC-TMAO and TNF-α that depended on TMAO-response group. These findings promote precision nutrition and have important ramifications for the eating behavior of adults who could benefit from reducing TMAO exposure, and for understanding factors that generate plasma TMAO.

## 1. Introduction

The circulating metabolome consists of metabolites that are absorbed directly from the diet, produced by human metabolism, and/or that are derived from the metabolism of the gut microbiome [1]. Studies of the plasma metabolome have played tremendous roles in identifying key microbiome-derived metabolites such as trimethylamine n-oxide (TMAO), short-chain fatty acids, tryptophan metabolites, and bile acids, that are associated with several diseases [2]. In addition to identifying disease drivers, metabolomic approaches have the potential to promote preventative care through individualized approaches such as precision nutrition, the practice of consuming a diet informed by factors including a person’s genetics, gut microbiome, and metabolic responses to foods. One tool to identify individual metabolic responses to foods is a mixed macronutrient tolerance test (MMTT), a standardized meal or shake that tests metabolic flexibility or the ability to switch between lipid and carbohydrate metabolism [3]. MMTTs also provide baseline metrics to compare metabolic responses across groups or before and after a lifestyle intervention [4]. In healthy adults, combining the insights of the MMTT with metabolomics could reveal predictive small molecule markers of metabolic dysregulation before clinical symptoms appear. This proof-of-concept study assesses the postprandial response of the microbiome-derived metabolite trimethylamine n-oxide (TMAO) after consuming a high-fat MMTT with minimal TMAO precursors choline, phosphatidylcholine, L-carnitine, and betaine.

TMAO can be absorbed directly by consuming foods containing TMAO or it can be formed via the microbial metabolism of choline, phosphatidylcholine, L-carnitine, and betaine to trimethylamine gas (TMA) [5,6,7]. TMA is absorbed and oxidized predominately by hepatic flavin-containing monooxygenase 3 (FMO3) to TMAO, which is associated with cardiovascular disease, chronic kidney disease, and type 2 diabetes; it has also been demonstrated to alter cholesterol metabolism and to provoke inflammation and thrombosis [6,8,9,10,11]. It is important to understand changes in postprandial TMAO concentrations and to assess whether total postprandial exposure to TMAO is associated with CVD risk factors in generally healthy adults. 

To investigate whether dietary components influence plasma TMAO concentrations, researchers have compared foods with different TMAO precursors (e.g., eggs containing phosphatidylcholine versus meat containing carnitine) [12], foods with the same TMAO precursor in different food matrices (e.g., eggs containing phosphatidylcholine versus soup supplemented with choline-bitartrate) [13], or the same TMAO-precursor-containing food in different doses [14] or on different dietary backgrounds (e.g., eggs containing phosphatidylcholine on a low- versus high-fat diet) [15]. In aggregate, these studies provide context for how specific nutrients, the food matrix, and the diet pattern impact the precursor’s effect on TMAO concentrations. However, whether TMAO concentrations change in response to a high-fat MMTT with minimal TMAO precursors is unknown. 

Addressing this gap, we leveraged the cross-sectional Nutritional Phenotyping Study, which deeply phenotyped male and female adults aged 18-65 years and with a BMI of 18 to 44 kg/m^2^ who were unmedicated for cardiometabolic diseases [16]. Individuals from the Nutritional Phenotyping Study provided a fecal sample, consumed an MMTT (60% fat, 25% sucrose; 42% of a standard 2000 kilo calorie diet) containing limited TMAO precursors, and had their metabolic response recorded over 6 h. First, we hypothesized that the MMTT would result in postprandial changes in TMAO concentration due to the release of phosphatidylcholines and bile acids during digestion. Second, we assessed whether there were individualized response types (i.e., non-responder, fast-responder, or slow-responder) and whether the responses were related to the composition of the gut microbiome, FMO3 genotype, or the plasma metabolome. Third, we related response types to cardiometabolic biomarkers asking if fasted or total exposure to TMAO over the 6-h postprandial period better predicted metabolic dysregulation. Our results have important implications for precision nutrition, eating behavior, and for broadening our understanding of food sources considered TMAO precursors. 

## 2. Results

### 2.1. Participant and Mixed Macronutrient Tolerance Test Characteristics 

Participant characteristics are described in Table 1. Male (*n* = 49) and female (*n* = 48) participants were randomly selected from each sex-age-BMI bin (see Methods regarding the Nutritional Phenotyping Study’s recruitment strategy) from the Nutritional Phenotyping Study for postprandial metabolomics assessment. This resulted in studying the metabolism of 30 adults aged 18–33 years, 32 adults aged 34–49 years, and 35 adults aged 50–65 years. The present cohort was multi-ethnic but was mostly represented by participants who self-identified as White (67%). This was similar to the 2020 California Census (71.1% White) [17]. Average concentrations of blood chemistries fell within clinically healthy boundaries.

The composition of the standard mixed macronutrient tolerance test (MMTT) is provided in Table 2. The MMTT consisted of 72% pasteurized liquid egg white, 15% palm oil shortening, and 14% granulated white sugar. The macronutrient composition was approximately 60% fat, 25% carbohydrate, and 15% protein. Of the calories from fat, 29% were from saturated fatty acids, 22% were from mono-unsaturated fatty acids, and 5.5% were from poly-unsaturated fatty acids. The fatty acids were predominately from triglycerides, the major constituent of palm oil. The main carbohydrate was sucrose. The MMTT contained limited amounts of choline (2.94 mg) and betaine (0.755 mg). The amount of choline was approximately 0.69% and 0.53% of the Adequate Intake for adult females and males, respectively. Carnitine was not an ingredient used in the MMTT.

### 2.2. Plasma TMAO Concentration Changes in Response to an MMTT

**Figure 1:**
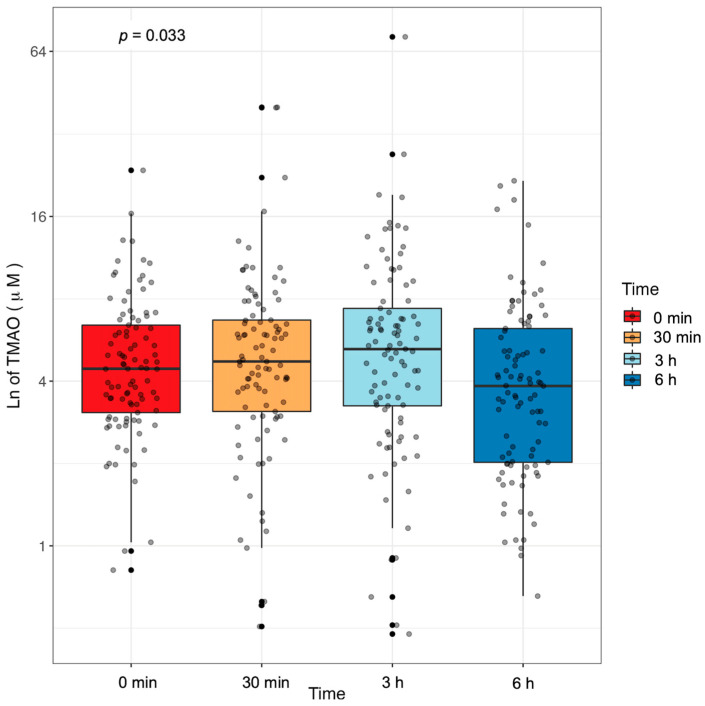
Plasma TMAO concentrations respond to an MMTT. Plasma TMAO concentrations in 97 adults change after consuming a mixed macronutrient tolerance test (MMTT) that contained minimal TMAO precursors. The *p*-value was calculated using ANOVA.

We first assessed whether plasma TMAO concentration responded to a mixed macronutrient tolerance test (MMTT) with limited amounts of TMAO-precursors in a cohort of adults unmedicated for cardiometabolic diseases (Figure 1). Fasting plasma TMAO concentration averaged 5.27 ± 3.43 µM, which increased 30 min after consuming the MMTT to 6.09 ± 6.01 µM (*p*-value = 0.244). Compared to fasting, average plasma TMAO remained elevated 3 h after consuming the MMTT (6.87 ± 8.11 µM, *p*-value = 0.038) and then significantly decreased from 3 h to 6 h (*p* = 0.011) to concentrations similar to baseline at 4.76 ± 4.00 µM. The average fold change was 4.43-fold, but individual responses were highly variable with the maximum fold change of 23 starting at 11 µM and decreasing to 0.46 µM.

**Figure 2:**
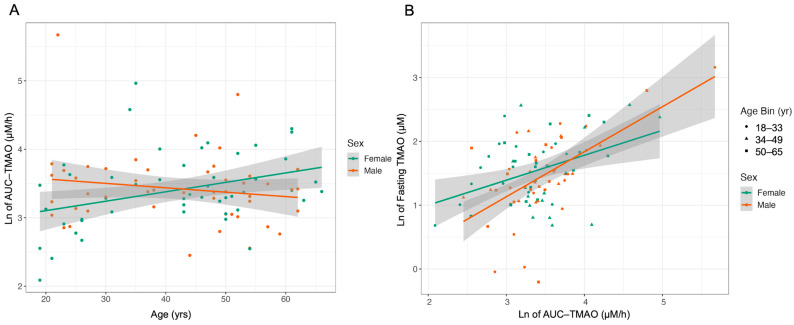
Postprandial TMAO exposure is different by sex and age. Exposure to plasma TMAO during a six-hour window following a high-fat, high-sugar MMTT differs with age in males and females and correlates to fasting TMAO plasma concentrations. (**A**) Exposure to TMAO increases with age in females but not in males (*p*-value = 0.0112). (**B**) Exposure to TMAO correlates with fasting TMAO concentrations in males and females but is higher in males (female, r = 0.41; male, r = 0.6).

Total area under the curve of TMAO concentration (AUC-TMAO) was calculated for each participant using the trapezoid rule. A significant sex by age interaction was related to AUC-TMAO (*p*-value = 0.0112) such that AUC-TMAO increased with age in females but not in males (Figure 2A). AUC-TMAO was not related to BMI (*p*-value = 0.419) nor fasting plasma cystatin C (*p*-value = 0.126), a marker of kidney function. This suggests that the observed changes in postprandial TMAO concentration are not driven by renal function. AUC-TMAO was correlated to fasting plasma TMAO concentration (r = 0.5), which was stronger in males (r = 0.6), than in females (r = 0.41) (Figure 2B).

### 2.3. Changes in TMAO Concentration after an MMTT Display Variation

**Figure 3:**
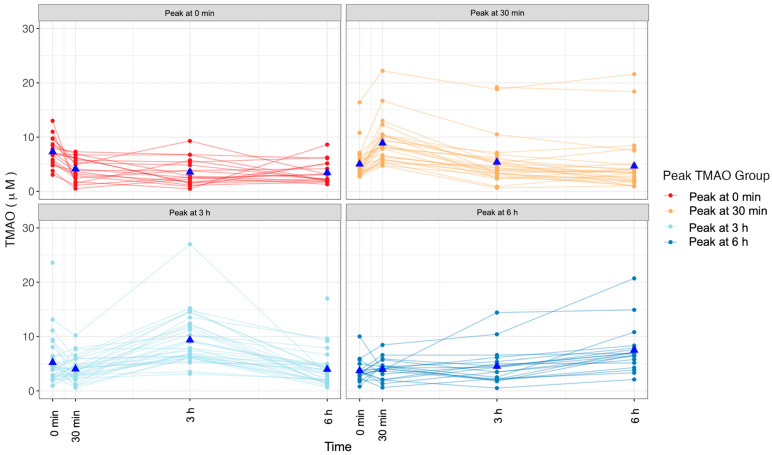
Postprandial TMAO concentrations are individualized. Variation in postprandial TMAO concentration was observed amongst participants. Participants were grouped by the timepoint (0 min, 30 min, 3 h, or 6 h) at which they experienced their maximal plasma TMAO concentration. Maximal TMAO concentrations were observed at each timepoint resulting in four TMAO-response groups. Each line represents an individual’s metabolic response. The blue triangles represent the group’s mean.

We noted individual variation in TMAO response to the MMTT. Although the average maximum TMAO concentration was recorded 3 h postprandially, the timing of the peak concentrations was variable by participant. We classified the participants according to the point where TMAO concentrations were maximally elevated (Figure 3). This resulted in four similar sized groups with 18 participants with maximum concentration at fasting (peak-0m), 27 with maximum concentration at 30 min (peak-30m), 33 with maximum concentration at 3 h (peak-3h), and 19 whose maximum concentration was observed at 6 h post-MMTT (peak-6h). These groups did not differ by sex (*p*-value = 0.597), age (*p*-value = 0.345), or BMI (*p*-value = 0.576). At a given blood draw, differences in plasma TMAO concentration by TMAO-response group were observed (Table 3). For example, in the fasted state, participants in the peak-0m group averaged a TMAO concentration of 7.29 ± 2.55 µM, which was significantly higher than that of individuals who experienced a delayed rise in TMAO concentration in the peak-6hr group, whose fasted concentration was 3.74 ± 1.97 µM (*p*-value = 0.015). Further, 30 min after the MMTT, the average TMAO concentration in the peak-30m group was 10.05 ± 7.13 µM, which was significantly higher than that in each of the other groups (*p*-value < 0.001). Three hours after the MMTT, participants in the peak-3h group had a significantly higher TMAO concentration than each of the other groups at 11.26 ± 11.88 µM.

Overall exposure to TMAO (AUC-TMAO) also varied by TMAO-response group. Participants whose maximal TMAO concentration was observed at 30 min or 3 h had the greatest quantified exposure to TMAO concentration over the 6 h window (*p* < 0.001). Overall, the four distinct response types allowed us to utilize a precision nutrition approach by asking if relationships between health parameters differed by TMAO response type.

### 2.4. Relationships between Fecal Microbiome and Postprandial TMAO Response Are Weak

After identifying distinct postprandial TMAO response types we interrogated the role of the gut microbiome using 16S rRNA sequence data isolated from stool collected prior to consuming the MMTT. We hypothesized that there could be compositional differences associated with differences in postprandial TMAO generation including its timed appearance and total amount (Supplemental Figure S1). To evaluate the effect of total TMAO generated, we related AUC-TMAO to α-diversity metrics. When controlling for sex and age, we identified non-statistically significant associations between Shannon diversity (β = 0.17, *p*-value = 0.077), Faith’s phylogenetic diversity (β = 0.06, *p*-value = 0.118), observed OTUs (β = 0.002, *p*-value = 0.172), Pielou’s evenness (β = 1.15, *p*-value = 0.16), and AUC-TMAO. In contrast, fasting TMAO concentration was significantly associated with Shannon diversity (β = 0.26, *p*-value = 0.0125) suggesting that postprandial changes in TMAO are likely driven by factors independent of gut diversity captured by 16S rRNA based analysis of microbial diversity in the stool.

We then assessed whether there were compositional differences between TMAO-response groups while controlling for sex and age. PERMANOVA analysis was assessed to test differences by TMAO-response group with relation to dissimilarity matrices calculated using weighted UniFrac, unweighted UniFrac, and Bray Curtis methods. With each statistic, no compositional differences were observed.

Choline TMA-lyase, the enzyme that utilizes choline and generates TMA is more abundant in Firmicutes than Bacteroidetes. The mean ratios of the relative abundances of Firmicutes to Bacteroidetes by individuals in the peak-0m, peak-30m, peak-3h, and peak-6h groups were 8.25 ± 11.44, 10.28 ± 12.94, 8.82 ± 8.94, and 6.70 ± 8.19 features, respectively, and were not statistically different. Similarly, abundances of Firmicutes were not significantly related to total exposure to TMAO in the 6 h window (*p*-value = 0.759).

Lastly, we assessed the relative abundance of microbes related to AUC-TMAO by dichotomizing the cohort using the median AUC-TMAO concentration of 29.25 µM/h. Differential abundance analysis via the DESeq2 likelihood ratio test revealed four genera that were more abundant in the below-median group (genera *prevotella, lactobacillus, catenibacterium,* and *dialister*) and two genera that were more abundant in the above-median group (genera *eggerthella* and *clostridium*) (Supplemental Table S1). In all cases, the actual abundances of these differentially abundant microbes were modest within the cohort and were present in ~20% (*lactobacillus* and *catenibacterium*) to 59% (*clostridium*) of participants.

### 2.5. FMO3 Genotype Does Not Relate to Postprandial TMAO Response

FMO3 oxidizes TMA to TMAO, and individuals with monogenic FMO3 variants may experience low-penetrance-associated predispositions such as increased plasma TMA concentrations [18]. We hypothesized that FMO3 genotype may affect postprandial TMAO concentrations by delaying the metabolite’s appearance in plasma. We genotyped two SNPS, rs2266782 (G>A, p.Glu158Lys) and rs2266780 (A>G, p.Glu308Gly), located on human chromosome 1 at 171 Mbp (GRCh38.p13). Both SNPs are missense variants in FMO3 exons (rs2266782, exon4; rs2266780, exon 7; and NP_008825.4) and are considered pathogenic; however, some reports state that the clinical significance is minor. The distribution of each genotype in our cohort is provided in Table 4 and shows that the SNPs were frequently in linkage disequilibrium. The minor allele frequencies of both SNPs were like global averages across populations reported by the National Center for Biotechnology Information [19,20]. The allele frequencies tended to most resemble populations of European descent as expected with the predominately White cohort. The minor allele frequency of rs2266782 was 41.75% (TwinsUK, 40.78%; TOPMED, 40.00%) and 20.10% for rs2266780 (Twins UK, 19.47%; TOPMED, 12.40%). Both SNPs were in Hardy Weinberg equilibrium (rs2266782, Chi square = 1.11, *p*-value = 0.289; rs2266780, Chi square = 0.05, *p*-value = 0.820).

**Figure 4:**
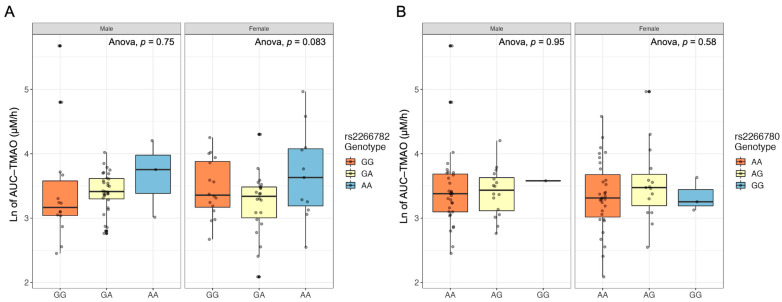
Relationship between postprandial TMAO exposure by genotype and sex. Relationship between AUC-TMAO (µM/h) by genotype at two FMO3 SNPs subset by sex. (**A**) Relationship between genotype at SNP rs2266782 (G>A) and AUC-TMAO. (**B**) Relationship between genotype at SNP rs2266780 (A>G) and AUC-TMAO. *p*-values were calculated using ANOVA.

When evaluating the relationship between genotype and AUC-TMAO, we observed a trend between increased concentration with variant alleles (Figure 4A,B), but neither SNP reached significance (rs2266782, *p*-value = 0.120; rs2266780 *p*-value = 0.824). Although not statistically significant at SNP rs2266782 (G>A) (*p*-value = 0.12), the median (interquartile range) of AUC-TMAO for genotypes GG, GA, and AA increased to 26.41 (21.37–40.66), 29.45 (22.99–35.00), and 40.20 (23.55–59.46) µM/h, respectively. Likewise, no significant differences by genotype were identified for SNP rs2266780 (A>G). The median concentration for individuals with the AA genotype was 28.37 µM/h (21.49–40.13) while it was 32.30 µM/h (22.43–38.48) for individuals with the AG genotype and 30.88 µM/h (25.11–36.33) for individuals with the GG genotype.

We stratified this analysis by biological sex and observed non-significant differences by genotype in both sexes. However, median AUC-TMAO tended to increase more prominently with the variant allele in males than females. In males at SNP rs2266782 (G>A), the median (interquartile range) of AUC-TMAO was 23.76 (20.93–36.23), 30.34 (27.07–37.13), and 42.66 (31.52–54.79) µM/h with genotypes GG, GA, and AA (*p*-value = 0.752). Similarly, in males at SNP rs2266780 (A>G), the median AUC-TMAO increased with copies of the variant G allele but did not reach statistical significance (*p*-value = 0.948). Males with the genotype AA had a median AUC-TMAO of 29.35 (22.15–39.83) µM/h, compared to 31.07 (22.54–37.70) and 35.86 (35.86–35.86) µM/h for AG and GG males. In females, the effect of genotype varied and neither SNP reached statistical significance (rs2266782, *p*-value = 0.083; rs2266780 *p*-value = 0.576). At rs2266782 (G>A), GG females had similar AUC-TMAO as females with the GA genotype at 28.69 (23.81–48.43) and 28.18 (20.20–32.55) µM/h, which was less than females with the AA genotype at 37.74 (24.33–58.94) µM/h. When we tested a dominant model such that one G allele could rescue the wildtype phenotype, the trend approached statistical significance (*p*-value = 0.070). At rs2266780 (A>G) in females, median AUC-TMAO values included 27.47 (20.46–39.64) µM/h for the AA genotype, 32.30 (24.50–39.81) µM/h for the AG genotype, and 25.89 (24.33–31.82) µM/h for the GG genotype.

Because the SNPs were often in linkage disequilibrium, we next assessed the effect of allelotype, or genotype at both SNPs simultaneously (Supplemental Figure S2A). In the whole cohort, no significant differences were observed. However, in the sex-stratified analysis, a relationship between FMO3 allelotype and AUC-TMAO was identified in females (*p*-value = 0.0476). Tukey’s post hoc test revealed marginal differences as females with the genotype rs2266782(G>A)-rs2266780(A>G) AA-AA had higher AUC-TMAO than those with the genotype GA-AA (*p*-value = 0.075). No associations were observed in males.

### 2.6. The Plasma Metabolome Differs by TMAO-Response Group

Biliary phosphatidylcholines are released to aid in lipid digestion and chylomicron production [21]. Knowing that the MMTT contained minimal TMAO precursors, we hypothesized that metabolites derived from host digestion, such as phosphatidylcholines and lysophosphatidylcholines, may relate to postprandial TMAO concentrations. We also hypothesized that bile acids might relate to postprandial TMAO concentrations because the bile acid receptor farnesoid X receptor (FXR) upregulates hepatic FMO3 expression [11]. Lastly, we assessed the metabolites p-cresol SO4, indSO4, and kynurenine because they are microbial products of protein foods and considered uremic toxins when renal function is poor, similar to TMAO. In all, we leveraged metabolomic data containing 73 phosphatidylcholines, 14 lysophosphatidylcholines, 14 bile acids, and 3 microbially derived metabolites. A complete list of the metabolites analyzed are provided in Supplemental Table S2.

**Figure 5:**
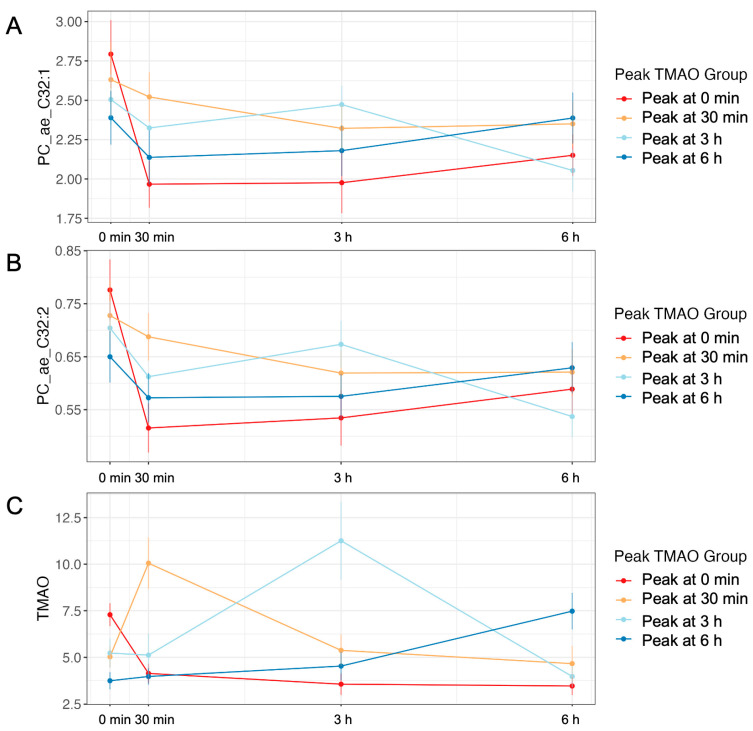
Postprandial responses of metabolites by peak TMAO-response group. Phosphatidylcholine with acyl-alkyl residue sum C32:2 (PC_ae_32:2, **A**) and phosphatidylcholine with acyl-alkyl residue sum C32:1 (PC_ae_C32:1, **B**) were positively correlated with TMAO (r = 0.295, Padj = 0.004; r = 0.250, Padj = 0.013) and showed similar appearance patterns to TMAO (**C**).

To assess which metabolites followed a similar metabolic pattern to TMAO, we correlated the AUC of the a priori metabolites to AUC-TMAO. We selected the AUC variables because they represent all blood draws. Additionally, we reasoned that if the metabolites traveled in a similar pattern to TMAO then their AUC would be related. The complete correlation results are presented in Supplemental Table S3. Of the 104 metabolites queried, seven were significantly correlated to AUC-TMAO, including three phosphatidylcholines, one bile acid, and two microbial-derived metabolites (Table 5). Phosphatidylcholine with acyl-alkyl residue sum C32:2 (PC_ae_32:2) and phosphatidylcholine with acyl-alkyl residue sum C32:1 (PC_ae_C32:1) were positively correlated with TMAO (r = 0.295, Padj = 0.004; r = 0.250, Padj = 0.013) and showed similar appearance patterns to TMAO for most response groups and time points (Figure 5). Lysophoshpatidylcholine acyl C14:0 (r = −0.240, Padj = 0.018) and phosphatidylcholine diacyl C40:1 (r = −0.235, Padj = 0.021) were inversely related to TMAO (Supplemental Figure S3).

### 2.7. Postprandial Exposure to TMAO Relates to CVD Biomarkers

We previously assessed the relationship between fasting plasma TMAO concentration and CVD biomarkers including clinical chemistry markers and inflammatory markers in this generally healthy cohort [22]. We found a strong association between TMAO and fasting TNF-α but limited associations with other CVD biomarkers [22]. However, changes to a metabolite’s fasting concentration may represent mature disease states where the body can no longer regulate homeostatic concentrations. We hypothesized that assessing metabolic response to a meal perturbation may provide a more sensitive assessment of metabolic health, especially in early disease phases before clinical symptoms have presented. We first assessed the relationships between total exposure to TMAO and fasting CVD biomarkers such as plasma glucose, triglyceride, cholesterol, and inflammatory markers in all 97 subjects. As previously identified, fasting TNF-α was the only biomarker that was significantly related to AUC-TMAO (β = 0.30, *p*-value = 0.044). Next, we expanded our analysis to assess whether exposure to plasma TMAO over the 6 h postprandial window related to total exposure to CVD biomarkers over the same window. Similar to assessments at the fasting time point, TNF-α remained the only statistically significant biomarker related to AUC-TMAO (β = 0.34, *p*-value = 0.024). This suggests that in healthy adults, fasting measurements of cardiometabolic biomarkers provide similar insights to characterizing metabolic responses of the biomarkers over a 6 h window.

**Figure 6:**
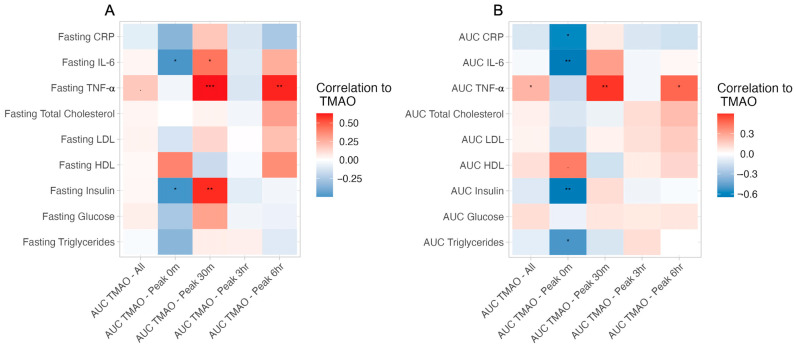
Relationships between cardiometabolic biomarkers and TMAO. Relationships between cardiometabolic biomarkers and exposure to TMAO (AUC-TMAO) by TMAO-response group identify similar relationships between fasted versus cumulative (AUC) concentrations. Assessing relationships by TMAO-response groups reveals relationships hidden when assessing the whole cohort. (**A**) The correlation between AUC-TMAO concentration and fasting cardiometabolic markers. (**B**) The correlation between AUC-TMAO and AUC-cardiometabolic markers. Red indicates a positive relationship and blue indicates a negative relationship. Correlations were calculated using the Spearman method. *** *p* < 0.001, ** *p* < 0.01, * *p* < 0.05, · *p* < 0.1.

Finally, in line with precision nutrition approaches, we assessed whether these relationships differed by TMAO response type. Interestingly, many differences by TMAO-response group were identified that were masked when assessing the cohort as one (Figure 6). Significant inverse relationships between AUC-triglycerides (β = −0.54, *p*-value = 0.002), AUC-insulin (β = −0.42, *p*-value = 0.008), AUC-interleukin 6 (AUC-IL6) (β = −0.44, *p*-value = 0.004), AUC-C-reactive protein (AUC-CRP) (β = −0.13, *p*-value = 0.029), and TMAO were identified in individuals in the peak-0m group. In contrast, individuals in the peak-30m group had a strong direct association with AUC-TNF-α (β = 0.8, *p*-value = 0.003), which was not present in the other groups. Together, our results suggest that postprandial TMAO dynamics may relate to different underlying health states in generally healthy adults.

## 3. Discussion

We present evidence that plasma TMAO concentration responds to a high-fat MMTT with minimal amounts of choline (2.94 mg; 0.69% and 0.53% of the Adequate Intake for adult females and males, respectively) and betaine (0.755 mg) in adults unmedicated for cardiometabolic diseases. We found that TMAO response to the MMTT was individualized such that the rate and total amount of TMAO generated ranged from rapid appearance and clearance of TMAO to slow. To understand what may be driving this phenomenon, we interrogated the roles of the fecal microbiome, the FMO3 genotype, and the plasma metabolome. Further, we assessed the relationship between postprandial exposure to TMAO and cardiometabolic biomarkers such as cholesterol, glucose, insulin, triglycerides, and inflammation.

In the healthy cohort, TMAO concentrations increased on average four-fold with postprandial concentrations averaging above 6 µM. Prospective literature consistently reports that in adults with cardiometabolic risk, fasting TMAO concentrations above approximately 5–6 µM are associated with increased cardiometabolic events over a multi-year period [23,24]. In healthy adults, TMAO is less prognostic with multiple reports finding no relation to disease [25,26]. Coronary artery calcium score, a marker of atherosclerosis, was not related to TMAO in adults in the CARDIA (Coronary Artery Risk Development in Young Adults) study suggesting a limited role for TMAO in early-stage atherosclerosis [25]. In disagreement, TMAO concentrations were found to incrementally affect future CVD development in a healthy cohort of generally healthy adults from Norfolk, United Kingdom [27]. In contrast to fasting concentrations, postprandial TMAO response may serve as an early indicator of CVD risk and have prognostic value for adults not displaying clinical signs of disease. Whether characteristics of transient TMAO responses like duration and magnitude affect the probability of CVD events has yet to be studied prospectively.

Our data demonstrated that individual’s TMAO response to a high-fat MMTT was highly variable. Assigning participants to a TMAO-response group based on the blood draw at which they experienced their maximal TMAO concentration, we observed four distinct groups. We sought to assess whether these groups were uniquely defined by their fecal microbiomes, their FMO3 genotype, or aspects of their plasma metabolome. Fasting TMAO concentration was significantly correlated to α-diversity metrics, but AUC-TMAO and TMAO-response group showed no relation. Further, compositional differences between response groups were also not identified. Overall, compositional differences assessed through diversity analyses were not associated with postprandial changes (duration and scale) in TMAO concentration. Fecal microbiome community abundance may be a poor tool for predicting the duration and magnitude of postprandial TMAO concentrations. Part of the explanation for this might be that the small intestine microbiome, which has the highest probability of encountering the MMTT during the postprandial window tested, may not be completely captured by the distal stool microbiota. Additionally, potential variations in gastric emptying and general gastrointestinal motility among participants could impact the “contact time” between the postprandial meal bolus and the incompletely captured small-intestine microbial community. The utility of the fecal microbiome in predicting fasting TMAO concentrations has been questioned previously. Ferrell et al. used metagenomics to determine the association between TMA-lyase, the bacterial enzyme that converts choline to TMA, and TMAO and found no relation, concluding that TMA-lyase gene count number is not a predictive marker of enzyme activity [28]. Given the difficulty of assessing acute changes in microbial gene expression in humans, that fecal sample collection times can vary even in controlled settings, and that the fecal microbiome may overly represent the lower colon, static 16S rRNA sequence assessment and metagenomic analysis may represent only a small component of the dynamic response to an MMTT.

We assessed two missense SNPs in exons of the FMO3 gene under the assumption that the SNPs influenced FMO3 activity. This assumption is supported by the literature relating the SNPs to decreased affinity to TMA, resulting in increases of the malodorous metabolite TMA [18]. We related FMO3 genotype to AUC-TMAO, hypothesizing that variant carriers may produce less TMAO over the 6 h window. We also tested whether variant carriers were more likely to be late responders and identified no relationship between TMAO-response group and genotype. In males but not females, we observed non-significant trends at each SNP such that variant carriers had higher median TMAO generation. The SNPs displayed linkage disequilibrium, and when considered together, significant differences were observed in females and not in males. Expression of FMO3 was shown to be sexually dimorphic in liver samples collected from the Caucasian adults in the Human Liver cohort [11,29]. Further, a study in mice demonstrated that FMO3 is inhibited by testosterone and upregulated by estrogen [11]. Thus, our sex-specific trend is in line with existing knowledge regarding FMO3. It is unclear why the variant genotype tended (sometimes non-significantly) to relate to higher total TMAO exposure. Whether other FMOs compensate for decreased FMO3 activity should be assessed.

Phosphatidylcholines are released to aid in digestion and may serve as a source of choline. Over the 6 h window, we identified three phosphatidylcholines (PCs; two correlated positively and one negatively) and one negatively correlated lysophoshpatidylcholine (LPC) that were significantly related to plasma TMAO exposure. Interestingly, the concentrations of the PCs decreased from fasting to 30 min but then followed dynamics similar to TMAO such that individuals with early rises in TMAO concentration also experienced early rises in PC concentration. Other studies have identified non-metabolic links between PCs, LPCs, and TMAO. Untargeted metabolomics and RNA expression experiments in mice consuming a high-fat diet with or without the microbial TMA-lyase inhibitor iodomethylcholine (IMC) identified that TMA may be a key regulator of the microbiome’s circadian rhythm [30]. Pathway analysis revealed that inhibition of TMA profoundly altered lipid and lipoprotein metabolism. For example, the diurnal rhythm of PC (16:0/22:6), PC (18:1/18:1), and LPC (16:0) were inverted by TMA inhibition. Our observed postprandial TMAO response may be a biproduct of mechanisms tied to the circadian rhythm. A subsequent study in ad libitum-fed mice identified that hepatic FMO3 gene expression displayed diurnal regulation but FMO3 protein levels did not [31]. Although hepatic FMO3 is the predominate oxidizer of TMA, hepatic FMO1 and FMO2 have also been demonstrated to generate TMAO [11]. Further, FMO oxidation of TMA may occur in many other tissues, as reported by GTEx [32]. In the mouse ileum, FMO1 and FMO2 demonstrated diurnal expression, which was disturbed by a high-fat diet [33]. The individualized TMAO-response types may be driven by these larger system-level phenomena that were not captured by our assessments of the fecal microbiome or the FMO3 genotype.

To comprehensively evaluate the postprandial TMAO response, we evaluated bile acids, which play a critical role in lipid digestion, have been demonstrated to influence the composition of the gut microbiome, and are related to TMAO through the FXR receptor, which regulates FMO3 [11]. The primary bile acid cholic acid increased both FMO3 and TMAO concentration via activation of hepatic FXR in mice fed a high-fat, cholesterol containing diet supplemented with the bile acid [11]. In the present study, the secondary bile acid, taurine-conjugate lithocholic acid (TLCA) was associated with total TMAO concentration across the 6 h window. The presence of TLCA may serve as a signature of bile-acid-induced upregulation of FMO3 resulting in TMAO generation.

We also evaluated the microbial-derived metabolites kynurenine, indoxyl sulfate (indSO4), and p-cresol sulfate (p-cresolSO4). In addition to being associated with cardiometabolic diseases, the metabolites are often derived from similar animal-protein food sources as TMAO [34]. Kynurenine and indSO4 are derived from the microbial metabolism of the amino acid tryptophan, while p-cresolSO4 is derived from the amino acids phenylalanine and tyrosine. Total exposures of P-cresolSO4 and indSO4 were directly related to TMAO, suggesting that this microbial-derived signature is present in healthy states. In vitro studies could test the effect of this signature on inflammatory gene expression to investigate potential disease mechanisms. Additionally, this finding suggests microbial inhibitors may offer a compromise between consuming protein and limiting exposure to TMAO, indSO4, and p-cresolSO4 and could be tested. 

TMAO response to a high-fat MMTT may have important ramifications for eating behavior. Time restricted eating (TRE) is a dietary pattern with a window of unrestricted feeding followed by a longer window of fasting and considers aligning feeding with circadian rhythm [35]. TRE is considered a healthy eating pattern and has been demonstrated to facilitate weight loss and improve cardiometabolic biomarkers such as plasma lipids, glucose, and insulin [36]. As demonstrated in the present study, a 6 h postprandial window does not resolve all metabolites to fasting concentrations. Thus, eating frequently may result in a perpetuated postprandial state. Thus, other benefits of TRE may be limiting exposure to microbiome-derived metabolites, such as TMAO. Future longitudinal studies should assess exposure to postprandial TMAO concentration in a time restricted eating pattern compared to an unrestricted eating pattern.

In the generally healthy cohort, we identified a robust association between AUC-TMAO and TNF-α in the full cohort and in the peak-30m and peak-6h groups. The relationship between TMAO and inflammation has been described. In an atherogenic mouse model, LDL receptor knockout (LDLR^-/-^) mice given water supplemented with 1.3% choline displayed high plasma TMAO concentrations and upregulated expression of inflammatory genes including TNF-α [37]. In the same study, acute rises in TMAO concentration via an intraperitoneally injected sterile solution of TMAO resulted in increased phosphorylation of p65 NF-κβ protein levels in the aortas of LDLR^-/-^ mice. However, in LDLR^-/-^ mice treated with an antisense nucleotide to knockdown FMO3, TMAO levels were reduced but TNF-α expression remained significantly higher than that in the control [38]. The treatment resulted in the decreased expression of the transcription factors PPARα and KLF15, which have been related to inflammation and may explain the unexpected result. Interestingly, antisense-treated mice also experienced a reduced pool of bile acids and phosphatidylcholines. Overall, we report that the postprandial relationships among inflammation, bile acids, and TMAO are in line with previous findings in fasted mice.

We found that the relationship between exposure to TMAO (AUC-TMAO) and fasting versus AUC-cardiometabolic markers was similar. However, by assessing individuals by their TMAO response type, we observed group-specific relationships between AUC-TMAO and cardiometabolic markers that were hidden when assessing the whole cohort. For example, participants in the peak-0m group had inverse relationships with insulin and IL-6 identified in the sub-analysis. Assessing metabolism via MMTTs may reveal disease states that have not presented as clinical symptoms, which may make them extremely useful in preventative health care.

Our study is limited by the number of participants with postprandial plasma values and the time points and total duration postprandial metabolism were measured. Furthermore, postprandial metabolism is continuous, and our sampling scheme at 30 min, 3 h, and 6 h postprandially likely resulted in missing true peak concentrations in each subject. We observed that participants in the peak-30m and peak-3h groups had the highest exposure to postprandial TMAO over the 6-h interval, but we did not fully capture TMAO exposure in participants in the peak-6h group whose concentrations had not resolved to baseline levels. Further, quantifying TMA would have provided further insights to the kinetics of TMAO generation but was not measured. We chose to group participants by the blood draw at which they experienced their peak TMAO concentration because we were interested in capturing differences in metabolic responses. This strategy resulted in similar-sized groups that did not differ by sex, age, or BMI, which satisfied our objective; however, an unsupervised grouping strategy, such as hierarchical clustering, may have better captured metabolic response types. Additionally, our genotyping strategy only captured two characterized missense FMO3 SNPs that can be inherited independently. Other SNPs have been described to affect FMO3 activity and may have provided a more complete surrogate of FMO3 activity [39]. The cohort was composed of multiple ethnicities but was predominately composed of participants self-identifying as White, limiting the translatability of these results to populations with varying allele frequencies. We hypothesized that groups of metabolites such as phosphatidylcholines and lysophosphatidylcholines may serve as a choline source contributing to postprandial TMAO changes. However, we only observed significant relationships between a small subset of the a priori metabolites and TMAO making it difficult to conclude the importance of these metabolite classes. Lastly, our findings are observational and hypothesis-generating and require validation.

Overall, these results suggest that TMAO concentration changes in response to a high-fat MMTT. We assessed whether these changes related to the composition of the gut microbiome, the FMO3 genotype, and the plasma metabolome. We identified select phosphatidylcholines, bile acids, and uremic toxins that related to total TMAO exposure. We identified unique response types such as early or late responders and found that some metabolites followed similar patterns. We also used the response types to identify associations with CVD biomarkers that were hidden when assessing the whole cohort. We show that the strong correlation between TMAO and TNF-α persists in the postprandial state but depends on TMAO response type. Overall, these findings promote precision nutrition and have important ramifications for the eating behavior of adults who could benefit from reducing TMAO exposure, and for understanding factors that generate plasma TMAO.

## 4. Materials and Methods

### 4.1. Study Design

The Nutritional Phenotyping Study has been previously described in depth [4,16,22,40,41]. Male and female adults unmedicated for cardiometabolic disease were recruited equally by sex, age, and BMI. Relevant to the aims of the current study, participants provided a stool sample as close to their study day as possible (within 24 h or less), consumed a standardized evening meal, conducted a 12 h water-only fast, and arrived at the Western Human Nutrition Research Center around 7:00 AM on their study day. Participants provided a fasted blood sample and were given 5 min to consume a standardized mixed macronutrient tolerance test (MMTT) in a shake form. Participants were instructed to consume the entire MMTT, which was noted by the study staff. Participants remained mostly sitting in an inclined position during the duration of the study. Blood was collected at 30 min, 3 h, and 6 h after finishing the MMTT. Blood was immediately processed and stored at −80 °C until processing. A total of 393 participants were recruited, and 361 participants finished all components of the Nutritional Phenotyping Study. Of these participants, 104 were chosen for postprandial metabolomics and 97 had complete TMAO data and were included in these analyses. The selected participants were equally distributed across the sexes, three age-bins (18–33 y, 34–49 y, and 50–65 y), and three BMI-bins (18.5–24.99, 25–29.9, and 30–44 kg/m^2^) used during recruitment.

Ethnicity was self-reported from a questionnaire with 12 categories: Hispanic or Latino/a, Black or African American, Asian, East Asian, South Asian, Southeast Asian, Middle Eastern, American Indian or Alaska Native, Native Hawaiian or Pacific Islander, decline to respond and other. Eighty-eight of the ninety-seven participants reported one ethnicity, while seven people reported two ethnicities, three people reported three ethnicities, and one person declined to respond. Due to the scarcity of many groups, we categorized people who reported Middle Eastern, Pacific Islander, Native Indian, or Other as “Other”. We also categorized individuals who reported multiple Asian ethnicities as “Asian”.

### 4.2. Mixed Macronutrient Tolerance Test

A detailed protocol describing the reasoning, nutrient composition, and distribution of the MMTT has been published [4]. The MMTTs were created in batches of 7 servings and stored at −20 °C for six weeks or less until they were consumed. Participants were provided a standard 403 g portion of the MMTT in a shake format to consume in 5 min. It is estimated that ~95% of the shake was consumed due to residual material sticking to the vesicle. Water (60 mL) was provided with the shake and subsequently restricted until after the blood draw at 30 min. Deionized water was then provided ad libitum throughout the study. The nutrient composition was estimated using the Nutritional Data System for Research (NDSR 2014, University of Minnesota, Minneapolis, MN, USA). An evaluation from Covance Laboratories (Labcorp Drug Development, Princeton, NJ, USA) estimated that 380 g of the MMTT would equate to 840 k-calories.

### 4.3. Gut Microbiome

A detailed protocol describing how the stool sample was acquired, transported, processed, as well as how the DNA from the stool sample was isolated, amplified, and sequenced has been previously described [20,39].

The 16S sequencing data were processed using Qiime2 (v2019.10) and analyzed using RStudio (v4.1.0) [42]. In Qiime2 (v2019.10), Cutadapt was used to demultiplex and trim the sequences [43]. Trimmed sequences were identified using DADA2 [44] and classified by the Naive Bayes classifier using the GreenGenes database (v13_8) at the threshold of 99% pairwise identity. In R, diversity and differential abundance was assessed in relation to TMAO phenotypes. The R packages qiime2R (v0.99.12), phyloseq (v1.26.1), vegan (v2.5-6), and DESeq2 (v1.32.0) were used to import the Qiime2 output into R, to package the data, to assess diversity, and to assess differential abundance, respectively. Only sequences present in at least 5% of samples were retained and analyses were conducted at the genus level resulting in 52 genera from 24 families. Alpha diversity was assessed by calculating Shannon, Pielou’s evenness, and observed OTUs statistics in Qiime2. Dissimilarity matrices were calculated using the phyloseq distance function and multivariate homogeneity of variances was tested using the PERMDISP2 method via the betadisper function. Analysis of variance using distance matrices was tested via the function adonis2 from the library vegan with 999 permutations, and controlling for sex, age, and BMI with marginal effects. The likelihood ratio test was used in DESeq2 with the covariates age, sex, and BMI. The Firmicutes to Bacteroidetes ratio was calculated for each individual and averaged by TMAO-response group.

### 4.4. Plasma Metabolome

TMAO and the plasma metabolome were measured using tandem mass spectrometry on an API 6500 QTRAP (Sciex, Framingham, MA, USA) using the MxP Quant 500 kit (Biocrates, Innsbruck, Australia). Metabolites detected through flow infusion acquisitions were processed in the MetIDQ Oxygen, with peak area used for quantification. Metabolites detected through chromatographic acquisitions were imported into MultiQuant v 3.2 (Sciex) and peak integrations were manually curated. An in-house python script was generated to correct for acquisition ion mass truncation and output header inconsistencies between MetIDQ and MultiQuant. Fasting plasma was analyzed for 362 participants randomized onto five plates. Postprandial samples for a subset of 104 participants were randomized onto four additional plates. The analysis of a single subaliquot of NIST Standard Reference Material 1950: Metabolites in Human Plasma (Sigma-Aldrich, St. Louis, MI, USA) was processed on each plate along with method blanks, manufacturer provided quality control plasma dilutions, and 7-point calibration curves for a subset of metabolites. Replicate analyses of 22 fasting plasma samples were included on plate five to further assess analytical precision. Data was normalized to the median of four mid-range quality control (QC2) solutions analyzed on each plate prior to exportation from MetIDQ. Of the 630 metabolites evaluated, 381 passed all quality control measures. A total of 97 participants had complete TMAO data and were included in the study. For analytes passing all quality control parameters, the overall replicate precision for analytes with concentrations greater than the limit of detection (LOD) was ~30 ± 1% (mean ± SEM) for both intra- and inter-plate replicates, but ranged from 12 to 56% for metabolite classes, with bile acids, diacylglycerols, and indole derivatives showing the worst precision. Across NIST replicates, 6.1% of 380 metabolites were above the LOD and 1.9% of 313 metabolites with concentrations greater than the limit of quantification had replicate precision of <30%, respectively.

### 4.5. CVD Biomarkers

Human blood was collected at fasting (0 min), 30 min, 3 h, and 6 h after consuming the MMTT. Blood was transported from the collection room to the laboratory on ice, and immediately processed to plasma or serum. Samples were stored at −80 °C. Plasma chemistry including glucose, triglycerides, total cholesterol, HDL cholesterol, and LDL cholesterol were measured on the COBAS INTEGRA 400 Plus Analyzer (Roche Diagnostic Systems, Basel, Switzerland) following the manufacturer’s instructions. Insulin was measured on the COBAS E 411 (Roche Diagnostic Systems, Basel, Switzerland) following the manufacturer’s instructions.

Concentrations of the inflammatory markers have been described [22]. Tumor necrosis factor alpha (TNF-α), interleukin 6 (IL-6), and c-reactive protein (CRP) were assayed in EDTA plasma using an electrochemiluminescence-based detection platform with multiplexed immunoassays using the V-PLEX system according to the manufacturers specifications (Meso Scale Diagnostics LLC, Rockville, MD, USA). CRP was measured using the V-PLEX Vascular Injury Panel 1. TNF-α and IL-6 were measured using the V-PLEX Custom Human Biomarker Proinflammatory Panel 1. Samples were diluted 1:1000 when assessing CRP and 1:2 when assessing TNF-α and IL-6. To assess plate to plate variation, three levels of lyophilized controls were used. Mean concentrations of duplicate wells were used for analysis.

### 4.6. Total Exposure Quantification

To quantify participants total exposure to metabolites or clinical biomarkers, the area under the curve (AUC) was calculated on raw variables using the trapezoid rule [4]. A total of 97 participants had TMAO concentrations at all 4 blood draws while 7 participants were missing 1 blood draw. Metabolites missing more than 10 values were removed resulting in removing PC_ae_C38:1 (17.34%) and PC_aa_C38:1 (5.57%). Of the remaining data, one metabolite was missing five entries, one was missing three, four were missing two, eighty-three were missing one and fourteen were complete. Due to the infrequency and randomness of the missing data, missing values were replaced with the metabolite’s median concentration.

### 4.7. Postprandial TMAO Response Patterns

To capture differences in TMAO-related metabolism, TMAO response types were categorized by the blood draw at which the participant had their highest TMAO concentration. Four groups were established including “peak-0m”, “peak-30m”, “peak-3h”, and “peak-6h”.

### 4.8. FMO3 Genotype Assessment

DNA was extracted from whole blood using a PAXgene Blood DNA kit (Qiagen, Hillden, Germany) as previously described [40]. SNPs rs2266782 (G>A, p.Glu158Lys, assay ID c_2461179_30) and rs2266780 (A>G, p.Glu308Gly, assay ID C_2220257_30) were genotyped using TaqMan Drug Metabolism Genotyping Assays and real time PCR using an Applied Biosystems QuantStudio 7 Flex Real-Time PCR System following the manufacturer’s instructions (ThermoFisher Scientific, Waltham MA, USA). QuantStudio Real-Time PCR software was used to determine the genotypes (ThermoFisher Scientific). Hardy-Weinberg equilibrium was tested using the HWChisq function in the R package HardyWeinberg (v1.7.5). Relationships between genotype and continuous variables were tested without assuming an inheritance pattern (i.e., categorically coded) unless otherwise stated.

### 4.9. Statistical Analysis

Statistical analyses were conducted in R Studio (v4.1.0). Before calculating parametric statistics, the variable’s distribution was tested using the Shapiro Wilk test. Variables were considered normally distributed if their W-statistic was greater than 0.95. Non-compliant variables were transformed via the natural log. Relationships between continuous variables were assessed using linear regression and controlled for a sex-by-age interaction. The sex-by-age interaction was assessed with type 2 sums of squares from the R package car (v3.1-0). Beta values are reported from natural log-transformed variables. Differences in the means between groups was assessed via the Student’s t-test or ANOVA followed by Tukey’s post-hoc test. A chi-square test was used to assess the relationship between the TMAO-response groups and categorical variables. Correlations were completed via the Spearman method. Significance was set at *p*-value < 0.05. For questions with multiple comparisons, false positives were controlled using the Benjamini and Hochberg (false discovery rate) method at a significance threshold of *p*-value < 0.05. Analyses were conducted on a MacBook Pro running MacOS Monterey software with 16 GB of memory.

## 5. Conclusions

Plasma TMAO responds to a high-fat MMTT with minimal amounts of choline and betaine. Variation in postprandial responses was observed such that some participants experience no, slow, or prolongated rises in TMAO concentrations. The composition of the gut microbiome had limited power to distinguish differences between response type as did FMO3 genotype characterized by two common SNPs. Distinct responses in the plasma metabolome were detected by TMAO-response group including correlations to select phosphatidylcholines and two secondary bile acids. TMAO response type was associated with the inflammatory marker TNF-α and TMAO-response group revealed hidden relationships to cardiovascular biomarkers. Assessing postprandial responses may be a better tool to predict cardiometabolic disease risk than values measured at fasting and should be assessed in healthy and diseased adults. Further, expanding foods that are considered TMAO precursors from choline, phosphatidylcholine, L-carnitine, and betaine to meals that produce a large release in biologically derived phosphatidylcholines like fatty meals should be considered. Lastly, eating behaviors, such as TRE, should be tested to reduce TMAO exposure and long-term CVD risk.

## Figures and Tables

**Table 1:** Descriptive statistics of the participants by sex. Blood parameters were measured in the fasting state.

	Male	Female	*p*-Value
*n*	49	48	
Age (yr)	41.86 (14.01)	41.71 (13.56)	0.958
BMI (kg/m^2^)	28.66 (5.52)	28.19 (5.41)	0.673
Ethnicity (%)			0.919
Caucasian	32 (65.3)	33 (70.2)	
Hispanic	9 (18.4)	5 (10.6)	
African American	1 (2.0)	2 (4.3)	
Asian	2 (4.1)	2 (4.3)	
Multiple	4 (8.2)	4 (8.5)	
Other	1 (2.0)	1 (2.1)	
Cystatin C (µM)	0.84 (0.14)	0.90 (0.14)	0.036
TMAO (µM)	5.41 (2.96)	5.12 (3.87)	0.679
Glucose (mg/dL)	92.93 (6.85)	95.80 (11.96)	0.152
Insulin (pM)	69.20 (42.79)	76.62 (100.28)	0.638
Triglycerides (mg/dL)	94.42 (38.55)	111.77 (60.86)	0.098
HDL-C (mg/dL)	58.58 (18.79)	44.66 (11.97)	<0.001
LDL-C (mg/dL)	107.65 (22.09)	106.86 (30.80)	0.885
Total Cholesterol (mg/dL)	175.11 (30.93)	164.51 (29.90)	0.091

Values are mean (SD) or count (%). *p*-values were tested using ANOVA. HDL-C, HDL-cholesterol; LDL-C, LDL-cholesterol.

**Table 2:** Composition of seven servings of the mixed macronutrient tolerant test (MMTT). Individual servings of 403 g were provided to participants to be consumed in a five-minute window.

Ingredient	Amount (g)	Weight %
Pasteurized liquid egg white	2105.9	70.20
Palm oil shortening	456.5	15.22
Granulated white sugar	421.4	14.05
Cellulose gum	8.3	0.28
Xanthan gum	4.7	0.16
Pure vanilla extract	1.0	0.03
Almond extract	1.0	0.03
Imitation butter flavor	1.0	0.03

**Table 3:** Plasma TMAO concentrations by TMAO-response group. Plasma TMAO concentrations (µM) at 0 min, 30 min, 3 h, and 6 h in individuals grouped by their TMAO response to an MMTT without choline, carnitine, or betaine.

	Peak 0 m	Peak 30 m	Peak 3 h	Peak 6 h	*p*-Value
*n* (Female)	18 (10)	27 (16)	33 (15)	19 (8)	0.597
Age (y)	36.67 (14.74)	42.67 (14.50)	42.36 (12.14)	44.37 (14.04)	0.345
BMI (kg/m^2^)	28.87 (4.80)	27.36 (5.45)	28.40 (5.98)	29.58 (5.14)	0.576
0 min	7.29 (2.55) ^A^	5.03 (2.92) ^A,B^	5.23 (4.35) ^A,B^	3.74 (1.97) ^B^	0.015
30 min	4.14 (2.14) ^A^	10.05 (7.13) ^B^	5.12 (6.61) ^A^	3.97 (1.90) ^A,C^	<0.001
3 h	3.56 (2.43) ^A^	5.37 (4.41) ^A,C^	11.26 (11.88) ^B^	4.53 (3.27) ^A^	0.001
6 h	3.46 (2.02) ^A^	4.66 (4.88) ^A,B^	3.97 (3.23) ^A^	7.48 (4.24) ^B^	0.006
AUC_TMAO_ (µM/h)	23.01 (9.01) ^A^	38.11 (29.46) ^B^	45.90 (46.71) ^B^	30.57 (16.58) ^A,B^	<0.001

Differences in the mean were calculated using ANOVA followed by a Tukey’s post-hoc test. Groups with significantly different mean values are indicated by different letters. Significance was set at *p* < 0.05.

**Table 4:** Distribution of FMO3 genotype and AUC-TMAO by genotype. Distribution of genotypes at two common SNPs in the FMO3 gene (**top**) and total AUC-TMAO (µM/hour) over the 6 h postprandial window (**bottom**). Values are counts (**top**) and means (standard deviation) (**bottom**).

		rs2266782, G > A (*n* = 97)			
**rs2266780, A > G (*n* = 97)**		GG	GA	AA	Total
	AA	30	26	6	62
	AG	0	26	5	31
	GG	0	1	3	4
	Total	30	53	14	97
		**rs2266782, G > A (µM/h)**			
**rs2266780, A > G (µM/h)**		GG (WT)	GA	AA	Average
	AA	42.01 (51.66)	28.17 (11.01)	48.54 (27.60)	39.57
	AG	NA	31.98 (11.63)	60.24 (51.92)	46.11
	GG	NA	35.86 (NA)	28.80 (7.90)	32.33
	Average	42.01	32.0	45.86	

**Table 5:** Correlation of AUC-TMAO and AUC metabolites. The relationship between 6 h exposure to phosphatidylcholines, lysophosphatidylcholines, bile acids, and uremic toxins was correlated to exposure to TMAO.

	r	P-Adj
PC_ae_C32.2	0.291	0.004
TLCA	0.265	0.009
PC_ae_C32.1	0.250	0.013
lysoPC_a_C14.0	−0.240	0.018
p-Cresol SO_4_	0.237	0.019
PC_aa_C40.1	−0.235	0.021
Indoxyl SO_4_	0.226	0.026
lysoPC_a_C16.0	−0.190	0.062
PC_ae_C40.2	0.176	0.085
PC_ae_C30.2	0.160	0.116
PC_aa_C40.2	−0.158	0.122
PC_ae_C40.5	0.145	0.155
lysoPC_a_C24.0	−0.141	0.167
lysoPC_a_C28.1	0.135	0.187
PC_ae_C30.0	0.134	0.191
TDCA	0.128	0.211
lysoPC_a_C18.0	−0.124	0.226
GUDCA	−0.123	0.230
TCDCA	0.122	0.236
PC_aa_C28.1	0.121	0.236

The top 20 most significant relationships are shown. Correlations are Spearman and *p*-values were adjusted using the BH method. TLCA, taurolithocholic acid; TDCA, taurodeoxycholic acid; TCDCA, taurochenodeoxycholic acid; GUDCA, glycoursodeoxycholic acid; PC phosphatidylcholine; ae, acyl-alkyl; aa, acyl-acyl; lysoPC, lysophosphatidylcholine.

## Data Availability

Requests for data from the USDA-ARS WHNRC Nutritional Phenotyping Study used in this analysis should be made via an email to the senior WHNRC author on the publication of interest. Requests will be reviewed quarterly by a committee consisting of the study investigators. Scripts used in statistical analyses are available at GitHub https://github.com/bytesizesci/FL100_TMAO_MealResponse, accessed on 10 January 2023).

## Supplementary Materials

The supporting information can be downloaded at: https://www.mdpi.com/article/10.3390/ijms24032074/s1.

## References

[1] Psychogios N., Hau D.D., Peng J., Guo A.C., Mandal R., Bouatra S., Sinelnikov I., Krishnamurthy R., Eisner R., Gautam B. (2011). The Human Serum Metabolome. PLoS ONE.

[2] Kim M., Huda M.N., Bennett B.J. (2022). Sequence Meets Function—Microbiota and Cardiovascular Disease. Cardiovasc. Res..

[3] Stroeve J.H.M., van Wietmarschen H., Kremer B.H.A., van Ommen B., Wopereis S. (2015). Phenotypic Flexibility as a Measure of Health: The Optimal Nutritional Stress Response Test. Genes Nutr..

[4] Newman J.W., Krishnan S., Borkowski K., Adams S.H., Stephensen C.B., Keim N.L. (2022). Assessing Insulin Sensitivity and Postprandial Triglyceridemic Response Phenotypes With a Mixed Macronutrient Tolerance Test. Front. Nutr..

[5] Wang Z., Klipfell E., Bennett B.J., Koeth R., Levison B.S., DuGar B., Feldstein A.E., Britt E.B., Fu X., Chung Y.-M. (2011). Gut Flora Metabolism of Phosphatidylcholine Promotes Cardiovascular Disease. Nature.

[6] Koeth R.A., Wang Z., Levison B.S., Buffa J.A., Org E., Sheehy B.T., Britt E.B., Fu X., Wu Y., Li L. (2013). Intestinal Microbiota Metabolism of L-Carnitine, a Nutrient in Red Meat, Promotes Atherosclerosis. Nat. Med..

[7] Taesuwan S., Cho C.E., Malysheva O.V., Bender E., King J.H., Yan J., Thalacker-Mercer A.E., Caudill M.A. (2017). The Metabolic Fate of Isotopically Labeled Trimethylamine- N -Oxide (TMAO) in Humans. J. Nutr. Biochem..

[8] Zhou Z., Jin H., Ju H., Sun M., Chen H., Li L. (2022). Circulating Trimethylamine-N-Oxide and Risk of All-Cause and Cardiovascular Mortality in Patients With Chronic Kidney Disease: A Systematic Review and Meta-Analysis. Front. Med..

[9] Zhu W., Gregory J.C., Org E., Buffa J.A., Gupta N., Wang Z., Li L., Fu X., Wu Y., Mehrabian M. (2016). Gut Microbial Metabolite TMAO Enhances Platelet Hyperreactivity and Thrombosis Risk. Cell.

[10] Zhuang R., Ge X., Han L., Yu P., Gong X., Meng Q., Zhang Y., Fan H., Zheng L., Liu Z. (2019). Gut Microbe–Generated Metabolite Trimethylamine *N* -oxide and the Risk of Diabetes: A Systematic Review and Dose-response Meta-analysis. Obes. Rev..

[11] Bennett B.J., de Aguiar Vallim T.Q., Wang Z., Shih D.M., Meng Y., Gregory J., Allayee H., Lee R., Graham M., Crooke R. (2013). Trimethylamine-N-Oxide, a Metabolite Associated with Atherosclerosis, Exhibits Complex Genetic and Dietary Regulation. Cell Metab..

[12] Cho C.E., Taesuwan S., Malysheva O.V., Bender E., Tulchinsky N.F., Yan J., Sutter J.L., Caudill M.A. (2017). Trimethylamine-*N*-Oxide (TMAO) Response to Animal Source Foods Varies among Healthy Young Men and Is Influenced by Their Gut Microbiota Composition: A Randomized Controlled Trial. Mol. Nutr. Food Res..

[13] Cho C.E., Aardema N.D.J., Bunnell M.L., Larson D.P., Aguilar S.S., Bergeson J.R., Malysheva O.V., Caudill M.A., Lefevre M. (2020). Effect of Choline Forms and Gut Microbiota Composition on Trimethylamine-N-Oxide Response in Healthy Men. Nutrients.

[14] Krishnan S., O’Connor L.E., Wang Y., Gertz E.R., Campbell W.W., Bennett B.J. (2021). Adopting a Mediterranean-Style Eating Pattern with Low, but Not Moderate, Unprocessed, Lean Red Meat Intake Reduces Fasting Serum Trimethylamine N-Oxide (TMAO) in Adults Who Are Overweight or Obese. Br. J. Nutr..

[15] Boutagy N.E., Neilson A.P., Osterberg K.L., Smithson A.T., Englund T.R., Davy B.M., Hulver M.W., Davy K.P. (2015). Short-Term High-Fat Diet Increases Postprandial Trimethylamine-N-Oxide in Humans. Nutr. Res..

[16] Baldiviez L.M., Keim N.L., Laugero K.D., Hwang D.H., Huang L., Woodhouse L.R., Burnett D.J., Zerofsky M.S., Bonnel E.L., Allen L.H. (2017). Design and Implementation of a Cross-Sectional Nutritional Phenotyping Study in Healthy US Adults. BMC Nutr..

[17] U.S. Census Bureau QuickFacts: California. https://www.census.gov/quickfacts/CA.

[18] Cashman J.R., Akerman B.R., Forrest S.M., Treacy E.P. (2000). Population-specific polymorphisms of the human fmo3 gene: Significance for detoxication. Drug Metab. Dispos..

[19] DbSNP Short Genetic Variants Rs2266782. https://www.ncbi.nlm.nih.gov/snp/rs2266782.

[20] DbSNP Short Genetiic Variants Rs2266780. https://www.ncbi.nlm.nih.gov/snp/rs2266780.

[21] Voshol P.J., Minich D.M., Havinga R., Elferink R.P.J.O., Verkade H.J., Groen A.K., Kuipers F. (2000). Postprandial Chylomicron Formation and Fat Absorption in Multidrug Resistance Gene 2 P-Glycoprotein–Deficient Mice. Gastroenterology.

[22] James K.L., Gertz E.R., Cervantes E., Bonnel E.L., Stephensen C.B., Kable M.E., Bennett B.J. (2022). Diet, Fecal Microbiome, and Trimethylamine N-Oxide in a Cohort of Metabolically Healthy United States Adults. Nutrients.

[23] Tang W.H.W., Wang Z., Levison B.S., Koeth R.A., Britt E.B., Fu X., Wu Y., Hazen S.L. (2013). Intestinal Microbial Metabolism of Phosphatidylcholine and Cardiovascular Risk. N. Engl. J. Med..

[24] Qi J., You T., Li J., Pan T., Xiang L., Han Y., Zhu L. (2018). Circulating Trimethylamine N-Oxide and the Risk of Cardiovascular Diseases: A Systematic Review and Meta-Analysis of 11 Prospective Cohort Studies. J. Cell. Mol. Med..

[25] Meyer K.A., Benton T.Z., Bennett B.J., Jacobs D.R., Lloyd-Jones D.M., Gross M.D., Carr J.J., Gordon-Larsen P., Zeisel S.H. (2016). Microbiota-Dependent Metabolite Trimethylamine N-Oxide and Coronary Artery Calcium in the Coronary Artery Risk Development in Young Adults Study (CARDIA). J. Am. Heart Assoc..

[26] Andraos S., Jones B., Lange K., Clifford S.A., Thorstensen E.B., Kerr J.A., Wake M., Saffery R., Burgner D.P., O’Sullivan J.M. (2021). Trimethylamine N-Oxide (TMAO) Is Not Associated with Cardiometabolic Phenotypes and Inflammatory Markers in Children and Adults. Curr. Dev. Nutr..

[27] Tang W.H.W., Li X.S., Wu Y., Wang Z., Khaw K.-T., Wareham N.J., Nieuwdorp M., Boekholdt S.M., Hazen S.L. (2021). Plasma Trimethylamine N-Oxide (TMAO) Levels Predict Future Risk of Coronary Artery Disease in Apparently Healthy Individuals in the EPIC-Norfolk Prospective Population Study. Am. Heart J..

[28] Ferrell M., Bazeley P., Wang Z., Levison B.S., Li X.S., Jia X., Krauss R.M., Knight R., Lusis A.J., Garcia-Garcia J.C. (2021). Fecal Microbiome Composition Does Not Predict Diet-Induced TMAO Production in Healthy Adults. J. Am. Heart Assoc..

[29] Schadt E.E., Molony C., Chudin E., Hao K., Yang X., Lum P.Y., Kasarskis A., Zhang B., Wang S., Suver C. (2008). Mapping the Genetic Architecture of Gene Expression in Human Liver. PLoS Biol..

[30] Schugar R.C., Gliniak C.M., Osborn L.J., Massey W., Sangwan N., Horak A., Banerjee R., Orabi D., Helsley R.N., Brown A.L. (2022). Gut Microbe-Targeted Choline Trimethylamine Lyase Inhibition Improves Obesity via Rewiring of Host Circadian Rhythms. eLife.

[31] Astafev A.A., Patel S.A., Kondratov R.V. (2017). Calorie Restriction Effects on Circadian Rhythms in Gene Expression Are Sex Dependent. Sci. Rep..

[32] GTExPortal. https://www.gtexportal.org/home/gene/FMO.

[33] Dantas Machado A.C., Brown S.D., Lingaraju A., Sivaganesh V., Martino C., Chaix A., Zhao P., Pinto A.F.M., Chang M.W., Richter R.A. (2022). Diet and Feeding Pattern Modulate Diurnal Dynamics of the Ileal Microbiome and Transcriptome. Cell Rep..

[34] Falconi C.A., Junho C.V.d.C., Fogaça-Ruiz F., Vernier I.C.S., da Cunha R.S., Stinghen A.E.M., Carneiro-Ramos M.S. (2021). Uremic Toxins: An Alarming Danger Concerning the Cardiovascular System. Front. Physiol..

[35] Vollmers C., Gill S., DiTacchio L., Pulivarthy S.R., Le H.D., Panda S. (2009). Time of Feeding and the Intrinsic Circadian Clock Drive Rhythms in Hepatic Gene Expression. Proc. Natl. Acad. Sci. USA.

[36] Moon S., Kang J., Kim S.H., Chung H.S., Kim Y.J., Yu J.M., Cho S.T., Oh C.-M., Kim T. (2020). Beneficial Effects of Time-Restricted Eating on Metabolic Diseases: A Systemic Review and Meta-Analysis. Nutrients.

[37] Seldin M.M., Meng Y., Qi H., Zhu W., Wang Z., Hazen S.L., Lusis A.J., Shih D.M. (2016). Trimethylamine N-Oxide Promotes Vascular Inflammation Through Signaling of Mitogen-Activated Protein Kinase and Nuclear Factor-κB. J. Am. Heart Assoc..

[38] Shih D.M., Wang Z., Lee R., Meng Y., Che N., Charugundla S., Qi H., Wu J., Pan C., Brown J.M. (2015). Flavin Containing Monooxygenase 3 Exerts Broad Effects on Glucose and Lipid Metabolism and Atherosclerosis. J. Lipid Res..

[39] Cashman J., Camp K., Fakharzadeh S., Fennessey P., Hines R., Mamer O., Mitchell S., Preti G., Schlenk D., Smith R. (2003). Biochemical and Clinical Aspects of the Human Flavin-Containing Monooxygenase Form 3 (FMO3) Related to Trimethylaminuria. CDM.

[40] Kable M.E., Chin E.L., Storms D., Lemay D.G., Stephensen C.B. (2021). Tree-Based Analysis of Dietary Diversity Captures Associations between Fiber Intake and Gut Microbiota Composition in a Healthy U.S. Adult Cohort. J. Nutr..

[41] Wang Y.E., Kirschke C.P., Woodhouse L.R., Bonnel E.L., Stephensen C.B., Bennett B.J., Newman J.W., Keim N.L., Huang L. (2022). SNPs in Apolipoproteins Contribute to Sex-Dependent Differences in Blood Lipids before and after a High-Fat Dietary Challenge in Healthy U.S. Adults. BMC Nutr..

[42] Bolyen E., Rideout J.R., Dillon M.R., Bokulich N.A., Abnet C.C., Al-Ghalith G.A., Alexander H., Alm E.J., Arumugam M., Asnicar F. (2019). Reproducible, Interactive, Scalable and Extensible Microbiome Data Science Using QIIME 2. Nat. Biotechnol..

[43] Martin M. (2011). Cutadapt Removes Adapter Sequences from High-Throughput Sequencing Reads. EMBnet.

[44] Callahan B.J., McMurdie P.J., Rosen M.J., Han A.W., Johnson A.J., Holmes S.P. (2016). DADA2: High-resolution sample inference from Illumina amplicon data. Nat. Methods.

