# Supplementary material for: Trimethylamine N-Oxide Response to a Mixed Macronutrient Tolerance Test in a Cohort of Healthy United States Adults
Source: Int J Mol Sci. 2023 Jan 20;24(3):2074. doi: 10.3390/ijms24032074 (PMC9917175; doi:10.3390/ijms24032074)
Supplement: Supplementary file 1 [file ijms-24-02074-s001.zip › IJMS_SupplementalTables_221206.pdf]

**Supplemental Table S1.** Differential abundance of genus level bacteria identified in participan

| hash ID | Base Mean | log2 Fold Change | lfcSE | Statistic |
|---|---|---|---|---|
| 098c3bbd8234f4ac198[illegible] | 2644.797543 | 0.636678934 | 2.919656061 | 126.4650425 |
| 803eb52cfe3d77bdf9fe | 3.71967458 | -1.595559731 | 1.252152435 | 43.60717769 |
| cbb17c39cfea0aa84321 | 19.58395687 | 2.053827386 | 2.93652842 | 21.01484623 |
| a3f9e91d15a189d0cf1[illegible] | 61.07314628 | -0.936009287 | 1.534221748 | 9.663043489 |
| a0798db0c868e9ad608 | 142.8544936 | 0.63230722 | 2.935199284 | 25.48475532 |
| 6960eba3db7d4d863d[illegible] | 47.066038 | 0.762509259 | 2.914439392 | 63.01935241 |

ts whose median AUC-TMAO was less than versus greater than 29.25 uM/hr. Differential abur

| P-value | Adj P-value | Kingdom | Phylum | Class |
|---|---|---|---|---|
| 2.43E-29 | 1.26E-27 | k__Bacteria | p__Bacteroidetes | c__Bacteroidia |
| 4.01E-11 | 6.96E-10 | k__Bacteria | p__Actinobacteria | c__Coriobacteriia |
| 4.56E-06 | 4.74E-05 | k__Bacteria | p__Firmicutes | c__Bacilli |
| 0.001880119 | 0.016294363 | k__Bacteria | p__Firmicutes | c__Erysipelotrichi |
| 4.46E-07 | 5.80E-06 | k__Bacteria | p__Firmicutes | c__Erysipelotrichi |
| 2.05E-15 | 5.32E-14 | k__Bacteria | p__Firmicutes | c__Clostridia |

ndance was performed with the log ratio test in DESeq2.

| Order | Family | Genus |
|---|---|---|
| o__Bacteroidales | f__Prevotellaceae | g__Prevotella |
| o__Coriobacteriales | f__Coriobacteriaceae | g__Eggerthella |
| o__Lactobacillales | f__Lactobacillaceae | g__Lactobacillus |
| o__Erysipelotrichales | f__Erysipelotrichaceae | g__Clostridium |
| o__Erysipelotrichales | f__Erysipelotrichaceae | g__Catenibacterium |
| o__Clostridiales | f__Veillonellaceae | g__Dialister |
