# Supplementary material for: Trimethylamine N-Oxide Response to a Mixed Macronutrient Tolerance Test in a Cohort of Healthy United States Adults
Source: Int J Mol Sci. 2023 Jan 20;24(3):2074. doi: 10.3390/ijms24032074 (PMC9917175; doi:10.3390/ijms24032074)
Supplement: Supplementary file 1 [file ijms-24-02074-s001.zip › IJMS_Sup_Figures_230119.pdf]

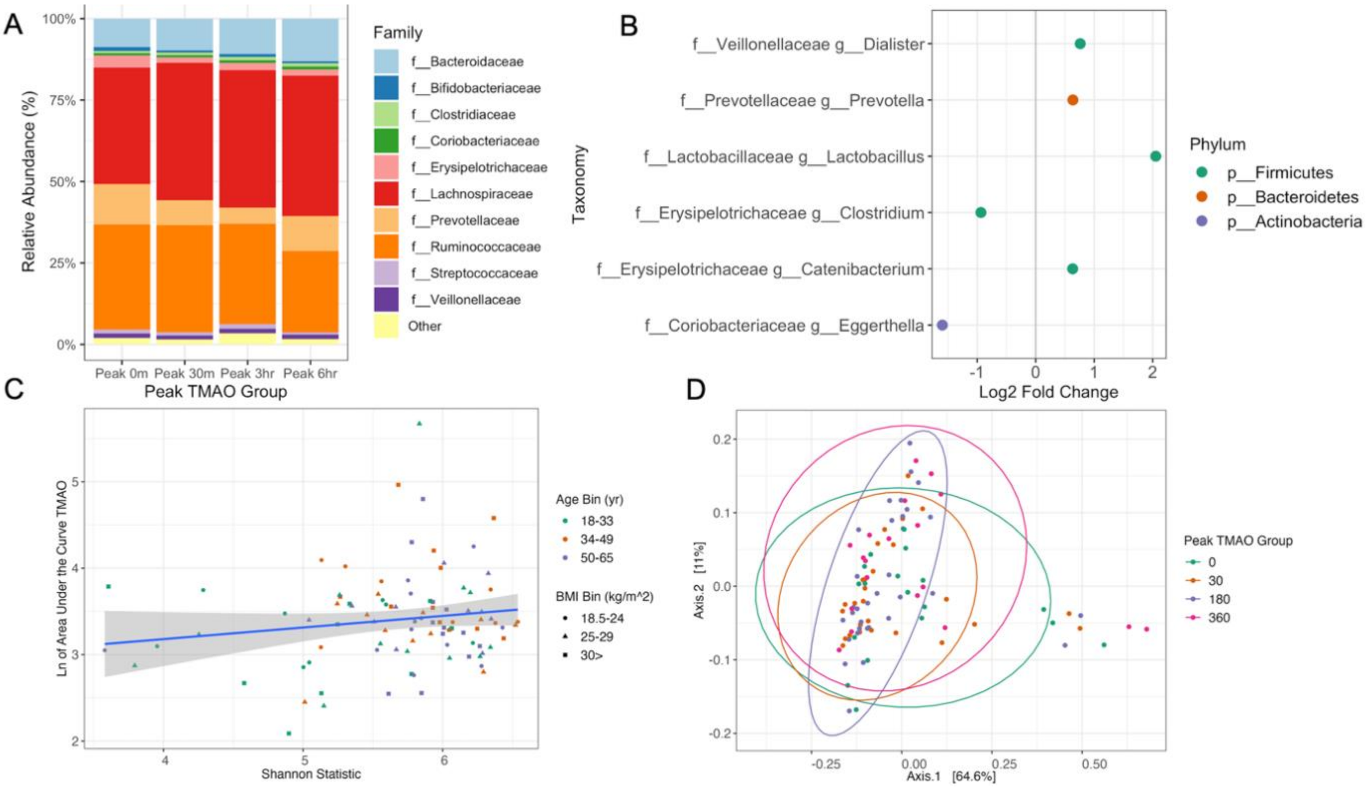

Figure S1. Compositional differences of the fecal microbiota related to TMAO exposure following a MMTT. (**A**) Family level relative abundance by peak-TMAO group. (**B**) Differential abundance in participants with AUC-TMAO concentration below or above the median of 29.25 μM/h (β = 0.17, p = 0.77). (**C**) Relationship between Shannon a-diversity and AUC-TMAO. (**D**) Weighted UniFrac b-diversity by peak-TMAO group shows non-significant differences.

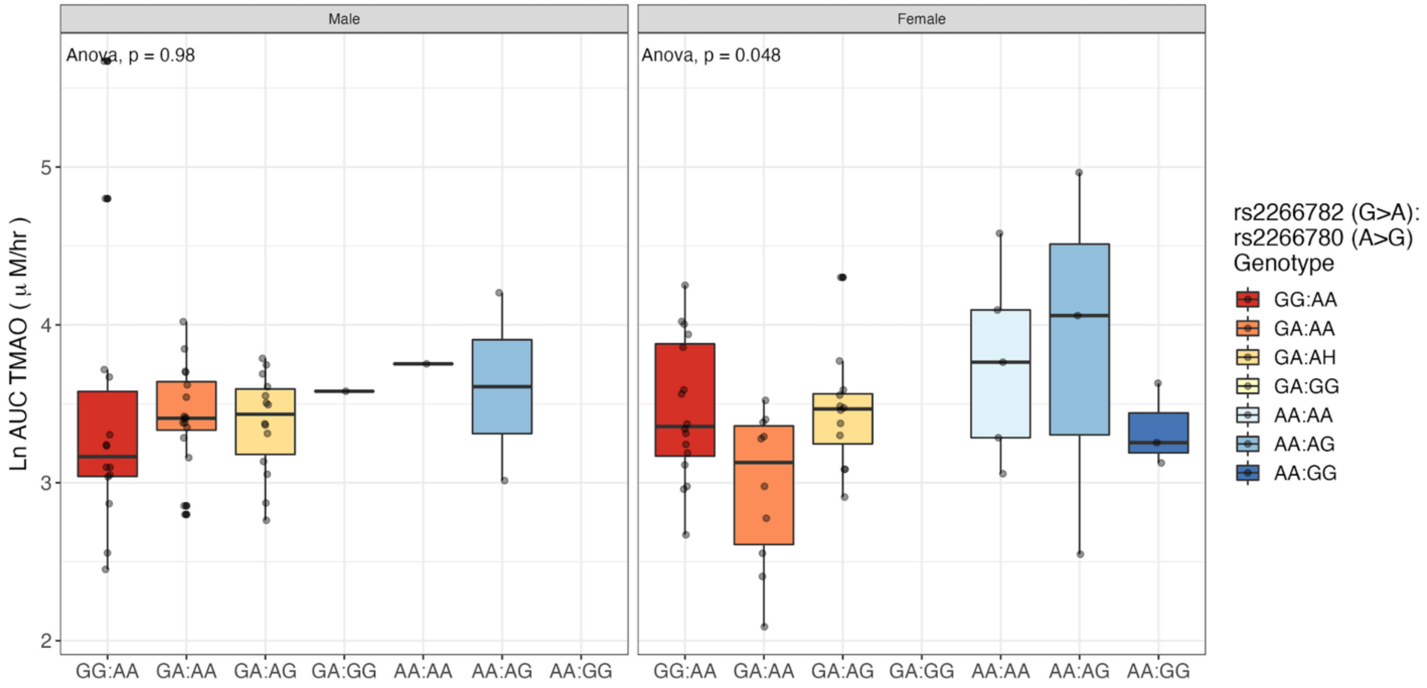

Figure S2. Relationship between *FMO3* allelotype and AUC-TMAO stratified by biological sex. Genotypes are presented by SNP rs2266782 (G>A):rs2266780 (A>G).

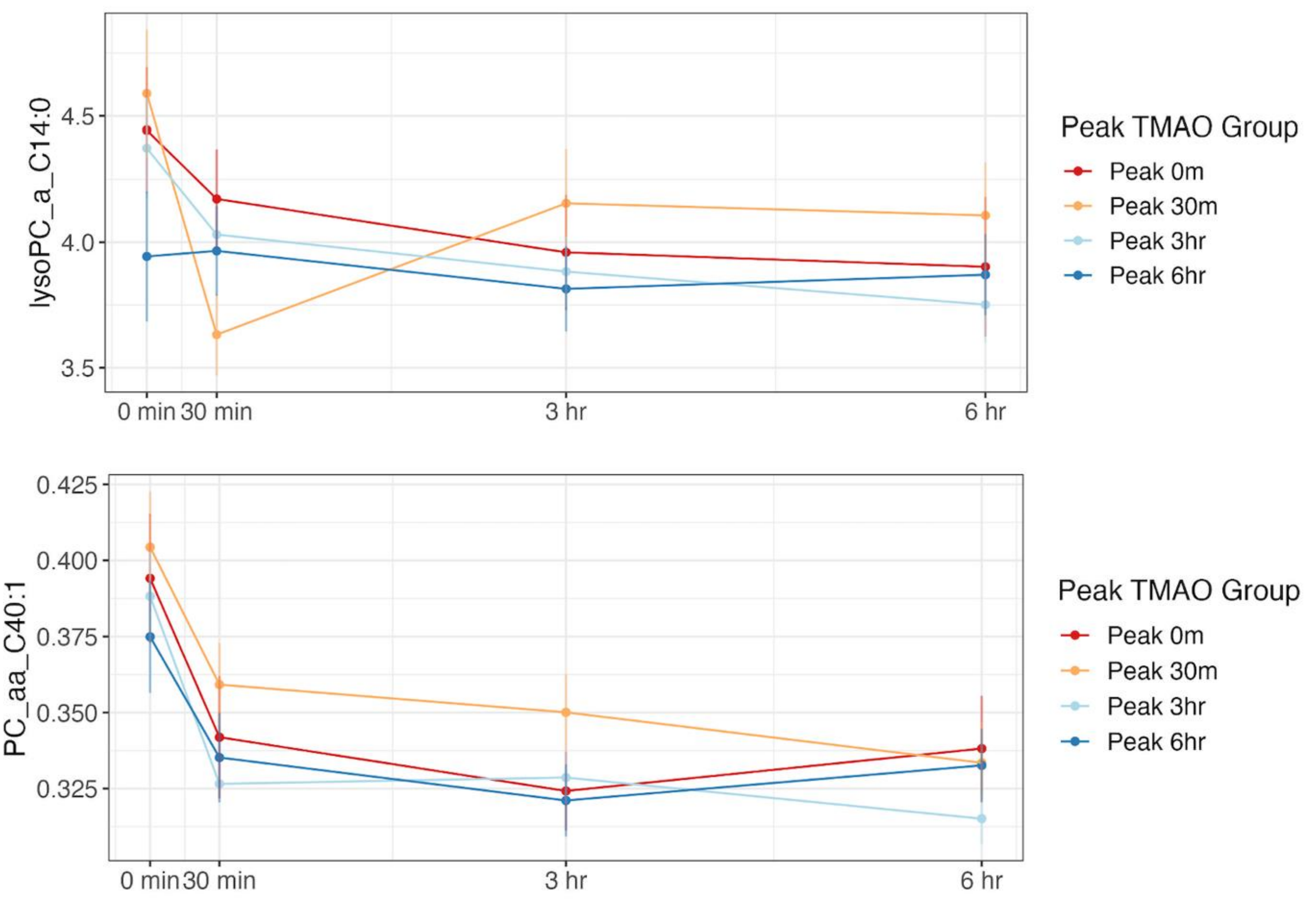

Figure S3. Postprandial responses of lysophosphatidylcholine acyl C14:0 (**top**) and phosphatidylcholine diacyl C40:1 (**bottom**) by peak-TMAO group.
